# *In vivo* targeting capacities of different nanoparticles to prostate tissues based on a mouse model of chronic bacterial prostatitis

**DOI:** 10.3389/fbioe.2022.1021385

**Published:** 2022-10-06

**Authors:** Ruimin Hu, Yang Yang, Guojing Song, Fuhan Zhao, Saipeng Chen, Zhansong Zhou, Jun Zheng, Wenhao Shen

**Affiliations:** Department of Urology, Southwest Hospital, Army Medical University (Third Military Medical University), Chongqing, China

**Keywords:** chronic bacterial prostatitis, targeting capacities, nanoparticles, prostate tissues, biodistribution

## Abstract

Chronic bacterial prostatitis usually occurs in men and seriously affects the quality of life of patients. The efficacy of chronic bacterial prostatitis treatment is limited by the difficulty for free drugs (e.g., antibiotics) to penetrate the prostate epithelium and target inflammatory tissues. The advent of nanotechnology offers the possibility to address this issue, such as the development of targeted nanoparticle delivery strategies that may overcome these important limitations. The physicochemical properties of nanoparticles, such as particle size, shape and surface modification ligands, determine their targeting effectiveness. In this study, nanoparticles with different physicochemical properties were prepared to explore and confirm their targeting capacities to inflammatory prostate tissues of chronic bacterial prostatitis, focusing on the effects of size and different modification ligands on the targeting performance. *In vivo* and *ex vivo* imaging results verified that folic acid-modified nanoparticles with a particle size of 180–190 nm via tail intravenous injection had the optimal targeting efficiency to prostate tissues. Our results provide an experimental basis and reference value for targeted therapy of prostate-related diseases with nanotechnology in the future.

## Graphical Abstract

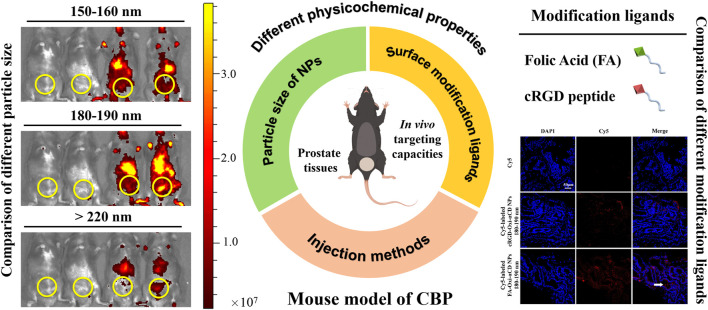

## 1 Introduction

Chronic bacterial prostatitis (CBP) accounts for 20% of chronic prostatitis and is a common disease in men (Halabi et al., 2020); CBP can cause many clinical symptoms, seriously affecting the quality of life of patients (Santharam et al., 2019). Recent studies have suggested that chronic prostatitis may be related to the occurrence and development of prostate cancer (Sfanos et al., 2018). As a result, increasing attention has been given to the disease, but there is still a lack of effective treatment. The effectiveness of CBP therapy depends on the ability of free drugs (e.g., antibiotics) to penetrate the prostate epithelium and reach the infected prostate tissues (Xiong et al., 2020). Unfortunately, it is difficult for most antibiotics to achieve the therapeutic effect in the prostate gland lumen due to their intrinsic physicochemical properties, such as lipid solubility, degree of ionization in plasma (pKa), protein binding, molecular radius and shape (Charalabopoulos et al., 2003). Only several antibiotics, such as fluoroquinolones and macrolides, can pass through the prostate epithelial barrier to reach the interior of the prostate lumen, but their efficacy descends obviously along with the increase of multiple drug resistance among bacteria (Su et al., 2020). There is an urgent need to find alternative agents to manage CBP.

Recently, nanodrugs have aroused a great interest in the treatment of antibacterial and inflammatory diseases (Wang et al., 2020, Wang et al., 2021a, Wang et al., 2021b; Cao et al., 2022; Ma et al., 2022; Wu et al., 2022). The advent of nanotechnology offers the possibility to address this issue, such as the development of targeted nanoparticle delivery strategies that may overcome these important limitations. Nanoparticles (NPs) are targeted to inflammatory lesions by passive or active targeting (Cao et al., 2020), which is mainly affected by their particle size, charge, shape, surface modification ligands and other physicochemical properties (Duan and Li, 2013; Zhao et al., 2019). Passive targeting mainly relies on the enhanced permeability and retention effect (EPR) (Golombek et al., 2018), and the passive accumulation of NPs at the lesion site is mainly determined by their particle sizes and shapes, with particle size being particularly important (Izci et al., 2021; Shukla et al., 2021). Active targeting is based on the overexpression of antigens, receptors and other biomolecules at the inflammatory sites (Attia et al., 2019). By modifying the surface of NPs with corresponding antibodies or ligands, they can specifically bind receptor molecules in the inflammatory area to achieve the goal of active targeting (Pearce and O’Reilly, 2019; Yoo et al., 2019).

It has been reported that a large number of activated M1 macrophages are infiltrated in inflammatory prostate tissues, and folic acid receptors (FRs) are highly expressed on the surface of activated M1 macrophages, which can specifically bind folic acid (FA) (Poh et al., 2018; Yang et al., 2021). Because FRs have comparable affinity for FA, FA-modified drugs or biomolecules can actively target cells or tissues that overexpress FRs (Muhamad et al., 2018). In our previous study (Zheng et al., 2022), it was confirmed that FRs were highly expressed in the prostate tissues of CBP mice, providing an experimental basis for the active targeting of FA-modified NPs. Studies have shown that neutrophils can be recruited to lesion sites by inflammatory factors due to their sensitivity to the inflammatory environment (Dorschner et al., 2020; Hou et al., 2022) and have a natural homing effect on prostatitis (Seo et al., 2014; Ahn et al., 2018; Safari et al., 2020). Therefore, the affinity of the cRGD peptide for the α_v_β_1_ integrin receptor (which is highly expressed on the neutrophil surface) (Hou et al., 2019) provides a theoretical basis for the active targeting of cRGD-modified NPs to inflammatory prostate tissues.

As mentioned above, the physicochemical properties of NPs, such as particle size and surface modification ligands, determine their ability to passively target and actively target *in vivo*. In a previous study (Zheng et al., 2022), our group first proved the feasibility of nanodrugs in the treatment of CBP, and verified the biosafety and effectiveness of nanodrugs at the cellular level and animal level, but the targeting capacities of NPs to inflammatory prostate tissues were not studied in depth. Since NPs penetrating the prostate epithelial barrier and achieving targeted delivery to prostate tissues are crucial to improve the efficacy of chronic prostatitis, it is of great significance to study the targeting capabilities of NPs with different physicochemical properties to prostate tissues, which may provide reference for targeted therapy of prostate-related diseases by nanotechnology in the future.

To verify the targeting efficiencies of NPs to inflammatory prostate tissues, we prepared NPs with different physicochemical properties, including comparison of three particle sizes (150–160, 180–190 and >220 nm) and two different surface modification ligands (FA and cRGD peptide), and the differences between the two different injection methods (tail intravenous and urethral injection) were discussed. Herein, NPs were fabricated by using ROS-responsive materials (4-(hydroxymethyl) phenylboronic acid pinacol ester-modified cyclodextrin, Oxi-αCD) that had good biocompatibility and were endowed with three different particle size distributions. NPs were externally decorated with polyethylene glycol (PEG) and FA or cRGD peptide to achieve long circulation and active targeting in the body. Both *in vivo* and *ex vivo* imaging experiments demonstrated that the accumulation of modified NPs in the prostate tissues was significantly increased. Among them, FA-modified NPs with a particle size of 180–190 nm via tail intravenous injection were superior in targeting efficiency.

## 2 Materials and methods

### 2.1 Materials

α-Cyclodextrin (α-CD) was provided by Tokyo Chemical Industry Co., Ltd. (Tokyo, Japan). 1-(3-Dimethylaminopropyl)-3-ethylcarbodiimide hydrochloride, 4-(hydroxymethyl) phenylboronic acid pinacol ester, 4-dimethylaminopyridine (DMAP), 1,1′-carbonyldiimidazole (CDI), tetrahydrofuran (THF), dimethyl sulfoxide (DMSO), acetone and Pluronic F127 were supplied by Sigma–Aldrich Co., Ltd. (Shanghai, China). 1,2-Distearoyl-sn-glycero-3-phosphoethanolamine-N-methoxy (polyethylene glycol)-2000 (DSPE-PEG_2000_), folic acid-conjugated DSPE-PEG-3400 (DSPE-PEG_3400_-FA) and cRGD-conjugated DSPE-PEG-2000 (DSPE-PEG_2000_-cRGD) were acquired from Xi’an Ruixi Biological Technology Co., Ltd. (Xi’an, China). Lecithin (refined) was supplied by Alfa Aesar (Shanghai, China). Cy5 free acid was purchased from Lumiprobe, LLC (Hallandale Beach, FL, United States). 4′,6-Diamidino-2-phenylindole (DAPI) was obtained from Beyotime Biotechnology Co., Ltd. (Shanghai, China). Oxi-αCD and Cy5-conjugated Oxi-αCD were synthesized according to our previously reported strategy (Wang et al., 2019). All chemicals were of analytical grade and used without further purification. Ultrapure water was used throughout this study.

### 2.2 Bacteria and animals

*Escherichia coli (E. coli)* isolates were obtained from patients with urinary infection at the First Affiliated Hospital of Army Medical University (Third Military Medical University), and *E2519* was selected to establish a murine model of chronic prostatitis (Zheng et al., 2022).

Male C57BL/6J mice (6–8 weeks old) were kept in an SPF-level sterile animal room and acclimatized for 1 week after arrival and were supplied by Hunan SJA Laboratory Animal Co., Ltd. (Changsha, China). All animal experiments were performed in accordance with the guidelines approved by the Laboratory Animal Welfare and Ethics Committee of Third Military Medical University (Chongqing, China).

### 2.3 Fabrication and characterization of Oxi-αCD NPs

Our previous work reported a self-assembly strategy to produce Oxi-αCD NPs (ROS-responsive drug carriers), which exhibited good biocompatibility and could be used as excellent drug delivery carriers (Zhang et al., 2015; Wang et al., 2019; Ni et al., 2020, Wang et al., 2021c.). Briefly, lecithin and DSPE-PEG_2000_ were dispersed in anhydrous ethanol and ultrapure water. The mixed solution was stirred gently at 65°C for 0.5 h. Subsequently, the organic solvent containing Oxi-αCD was added dropwise into the dispersed solution with 3 min of vortexing. The obtained mixture was cooled to 25°C and self-assembled for 2 h. Oxi-αCD NPs were harvested by centrifugation at 15000 rpm for 10 min, washed with 10 ml 5% F127 and 10 ml ultrapure water, and resuspended with 0.2 ml ultrapure water. FA-modified Oxi-αCD NPs (FA-Oxi-αCD NPs) and cRGD-modified Oxi-αCD NPs (cRGD-Oxi-αCD NPs) were synthesized in a similar procedure except that DSPE-PEG_2000_ and DSPE-PEG_3400_-FA or DSPE-PEG_2000_-cRGD were used. In addition, Cy5-conjugated Oxi-αCD was added to fabricate Cy5-labeled NPs. Herein, by adjusting the dose required for synthesis, the size of the NPs was controlled.

### 2.4 Murine model of CBP

10 μl of *E. coli E2519* (1×10^8^ colony forming units/mL) was injected into mice to establish a mouse model of CBP as described previously (Zheng et al., 2022). Mice were maintained with urinary retention under anesthesia for at least 0.5 h to facilitate bacterial culture. Moreover, 10 μl of PBS was inoculated into the control mice. The CBP mouse model was expectedly completed after 30 days of inoculation.

### 2.5 *In vivo* biodistribution study

CBP mice were randomly divided into 4 groups and were administered free Cy5, Cy5-labeled Oxi-αCD NPs, Cy5-labeled FA-Oxi-αCD NPs or Cy5-labeled cRGD-Oxi-αCD NPs (20 μg/100 μl of Cy5 per mouse in each group) via tail intravenous injection or urethral injection. In parallel, the control mouse group was injected with 100 μl of saline. After 2, 4, 8, 24 and 48 h of injection, mice were anesthetized, and fluorescence images of free Cy5 or Cy5-labeled NPs were observed using a live animal imaging system (Biolight Biotechnology Co., Ltd. Guangzhou, China) with a 625 nm excitation filter and a 680 nm emission filter. Furthermore, mice were euthanized at 24 and 48 h postinjection, and the prostates were collected and rinsed with PBS for *ex vivo* imaging. The fluorescence intensity was determined by a live animal imaging system. Subsequently, the prostates were collected for cryosection preparation (treated with 4% paraformaldehyde and DAPI staining) to detect the distribution of Cy5 or Cy5-labeled NPs in prostate tissues via confocal laser scanning microscopy (CLSM). All *in vivo* experiments were performed using 3 mice in independent experiments.

### 2.6 Statistical analysis

All results are expressed as the mean ± standard deviation (SD) of at least three independent experiments. Statistical analysis was conducted by GraphPad Prism software using one-way variance (ANOVA) with Tukey’s multiple comparison test for more than three groups and Student’s t test for two groups. For all statistical tests, *p* < 0.05 was considered to be statistically significant.

## 3 Results

### 3.1 Preparation and characterization of NPs

**FIGURE 1:**
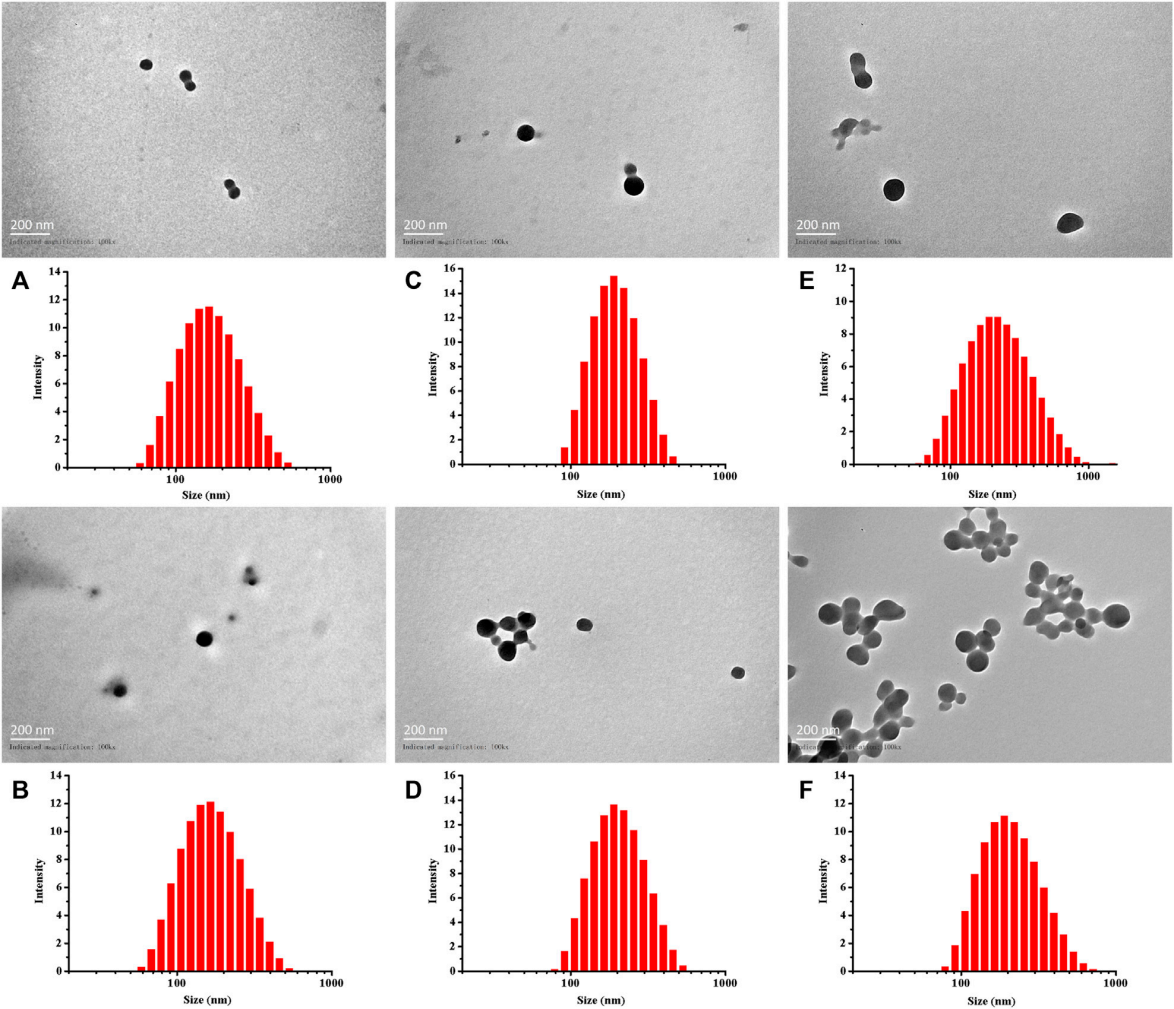
Size distribution and TEM images of **(A,C,E)** Cy5-labeled Oxi-αCD NPs and **(B,D,F)** Cy5-labeled FA-Oxi-αCD NPs: **(A,B)** 150–160 nm, **(C,D)** 180–190 nm, and **(E,F)** > 220 nm.

**FIGURE 2:**
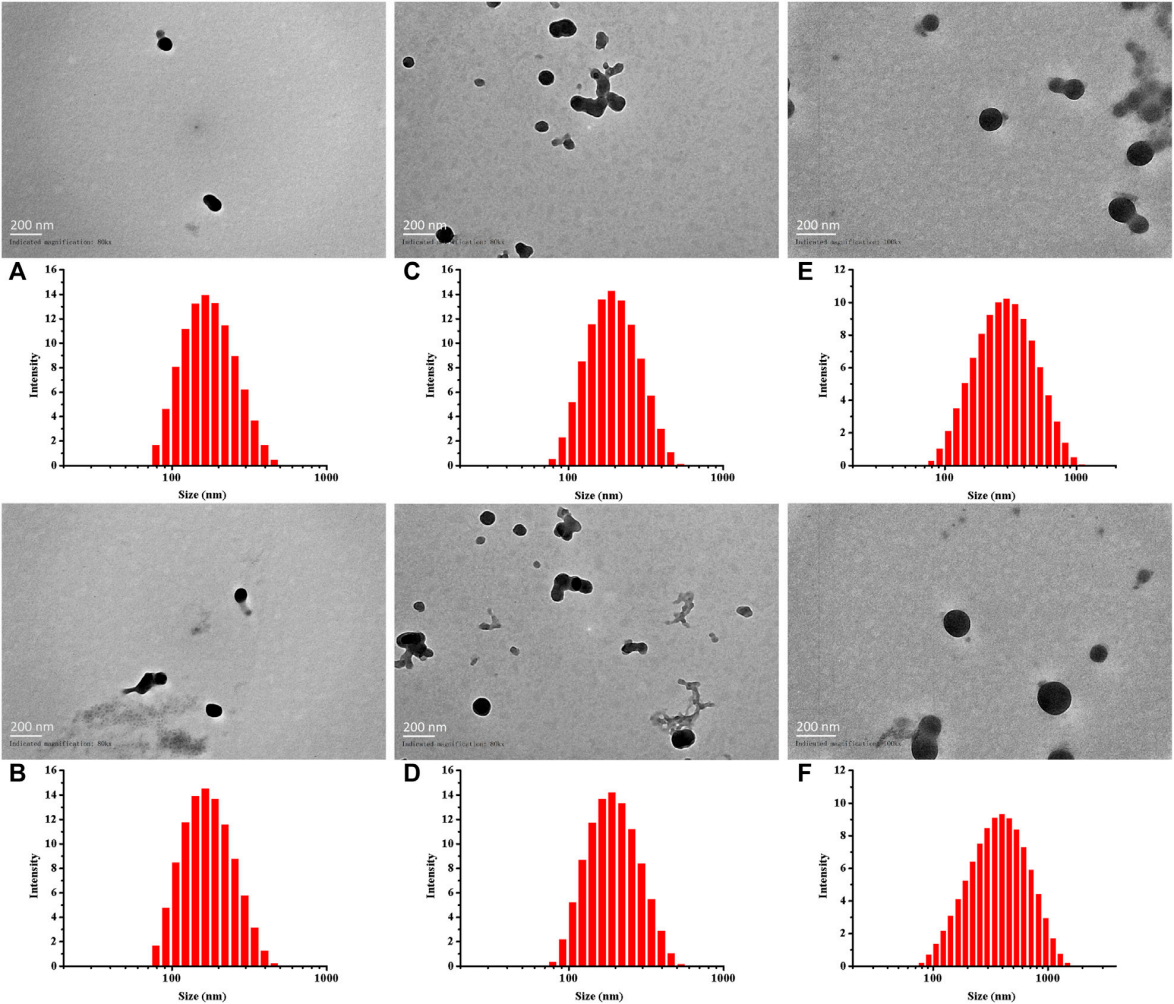
Size distribution and TEM images of **(A,C,E)** Cy5-labeled Oxi-αCD NPs and **(B,D,F)** Cy5-labeled cRGD-Oxi-αCD NPs: **(A,B)** 150–160 nm, **(C,D)** 180–190 nm, and **(E,F)** > 220 nm.

As previously described, by adjusting the dose required for synthesis, the particle sizes of Cy5-labeled Oxi-αCD NPs and Cy5-labeled modified Oxi-αCD NPs were controlled (Table 1). The physicochemical properties of the NPs are shown in Table 2. DLS measurements showed that Cy5-labeled Oxi-αCD NPs had average diameters of (a) 165, (c) 179 and (e) 226 nm, while Cy5-labeled FA-Oxi-αCD NPs were (b) 155, (d) 183 and (f) 226 nm, respectively. For cRGD modification, to roughly unify the synthesis conditions of nontargeted and targeted NPs, the formulations of nontargeted NPs were somewhat different from the abovementioned Cy5-labeled Oxi-αCD NPs in the FA group. The average sizes of Cy5-labeled Oxi-αCD NPs and cRGD-Oxi-αCD NPs were (a) 165, (c) 183, and (e) 269 nm and (b) 156, (d) 185, and (f) 332 nm, respectively. In addition, the zeta potential of NPs was not significantly altered by surface modification or particle size. Notably, all NPs exhibited a low polydispersity index (PDI), indicating a good distribution of these NPs, which can also be obtained from the DLS results. Through TEM images, we confirmed that the morphology of Cy5-labeled Oxi-αCD NPs, FA-Oxi-αCD NPs and cRGD-Oxi-αCD NPs was spherical and homogenous (Figure 1, Figure 2).

**TABLE 1:** Synthesis formula table of (A) (a, c, e) Cy5-labeled Oxi-αCD NPs and (b, d, f) Cy5-labeled FA-Oxi-αCD NPs; (B) (a, c, e) Cy5-labeled Oxi-αCD NPs and (b, d, f) Cy5-labeled cRGD-Oxi-αCD NPs.

(A)	Lecithin (mg)	DSPE-PEG_2000_ (mg)	DSPE-PEG_3400_-FA (mg)	Ethanol (ml)	Ultrapure water (ml)	Methanol (ml)	Another solvent (ml)	Oxi-αCD (mg)	Cy5-oxi-αCD (mg)
(a)	5.5	6.4	/	400	20	2	/		
(b)	5.2	4.0	4.0	400	20	2	/		
(c)	6.5	6.0	/	800	20	1.5	2 (THF)	45	5
(d)	6.6	4.1	4.0	800	20	1.5	2 (THF)		
(e)	6.5	6.2	/	800	20	1.5	2 (acetone)		
(f)	6.9	4.0	4.0	800	20	1.5	2 (acetone)		
**(B)**	**Lecithin (mg)**	**DSPE-PEG_2000_ (mg)**	**DSPE-PEG_3400_-cRGD (mg)**	**Ethanol (ml)**	**Ultrapure water (ml)**	**Methanol (ml)**	**Another solvent (ml)**	**Oxi-αCD (mg)**	**Cy5-Oxi-αCD (mg)**
(a)	5.2	5.9	/	400	20	2	/
(b)	4.9	5.0	5.0	400	20	2	/
(c)	6.7	9.0	/	200	5	1	2 (DMSO)	45	5
(d)	6.5	5.0	5.0	200	5	1	2 (DMSO)
(e)	6.0	6.0	/	200	5	1	2 (acetone)
(f)	4.0	5.0	5.0	200	5	1	2 (acetone)

**TABLE 2:** Size, PDI and zeta potential of (A) (a, c, e) Cy5-labeled Oxi-αCD NPs and (b, d, f) Cy5-labeled FA-Oxi-αCD NPs; (B) (a, c, e) Cy5-labeled Oxi-αCD NPs and (b, d, f) Cy5-labeled cRGD-Oxi-αCD NPs.

(A)	(a)	(b)	(c)	(d)	(e)	(f)
Size (nm)	165 ± 1	155 ± 2	179 ± 2	183 ± 2	226 ± 5	226 ± 2
PDI	0.24 ± 0.02	0.21 ± 0.01	0.16 ± 0.02	0.18 ± 0.01	0.34 ± 0.01	0.32 ± 0.01
Zeta potential (mV)	−27.8 ± 0.4	−28.2 ± 0.1	−20.2 ± 0.4	−22.2 ± 0.6	−21.8 ± 0.7	−20.9 ± 0.3
**(B)**	**(a)**	**(b)**	**(c)**	**(d)**	**(e)**	**(f)**
Size (nm)	165 ± 1	156 ± 2	183 ± 1	185 ± 2	269 ± 1	332 ± 4
PDI	0.17 ± 0.01	0.12 ± 0.01	0.18 ± 0.02	0.18 ± 0.01	0.27 ± 0.02	0.30 ± 0.02
Zeta potential (mV)	−24.2 ± 0.4	−25.3 ± 0.6	−23.6 ± 0.5	−22.7 ± 0.5	−25.3 ± 0.4	−28.6 ± 0.8

### 3.2 *In vivo* biodistribution of FA-modified NPs with different particle sizes

To investigate the targeting capabilities of NPs with different particle sizes to prostate tissues, *in vivo* biodistribution of Cy5-labeled NPs was detected in CBP mouse models by a living imaging assay. Since the prostate was too small to be accurately observed, we chose the entire lower urinary tract and parts of the reproductive system to study the targeting efficiency of NPs.

**FIGURE 3:**
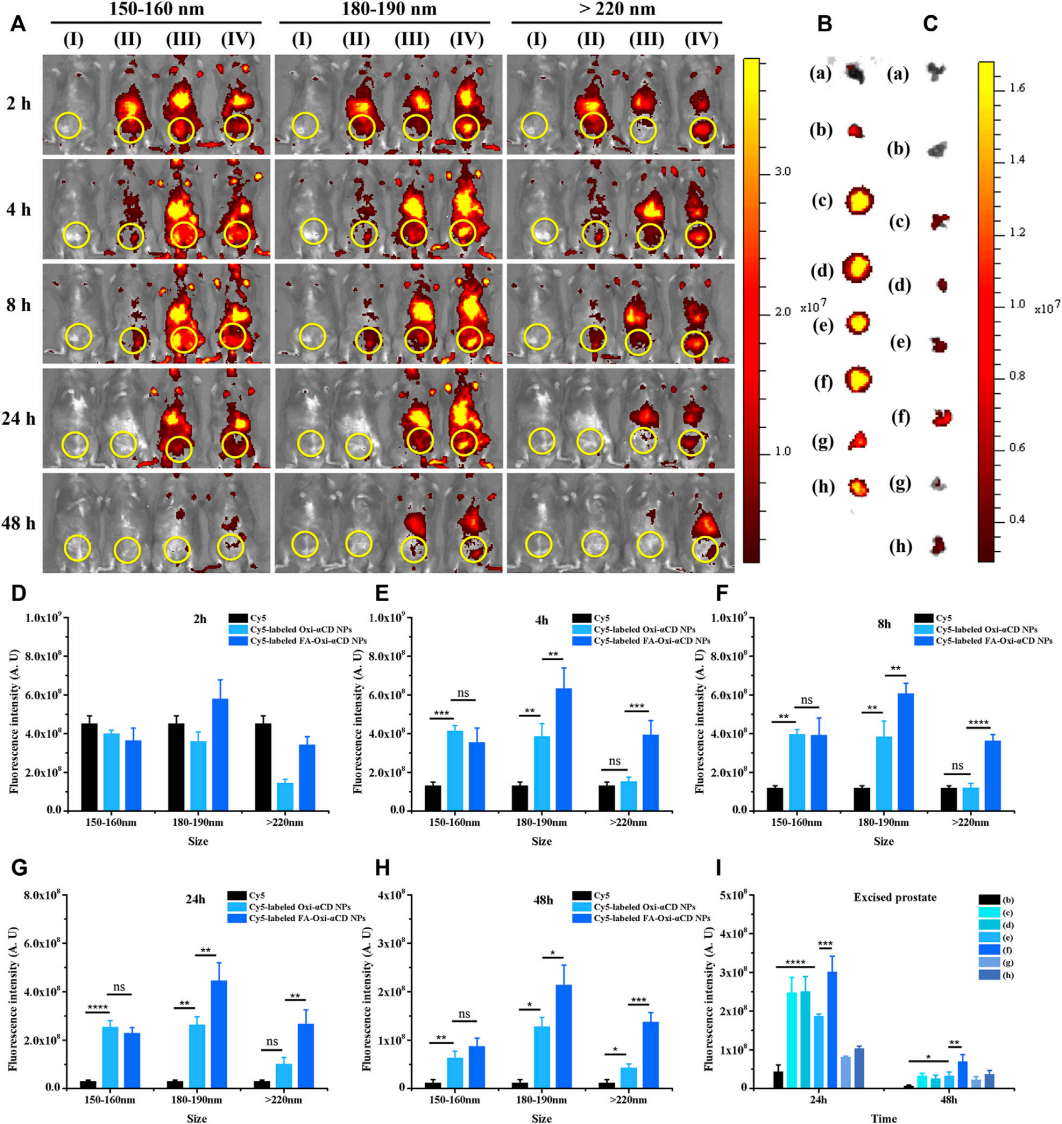
*In vivo* distribution of Cy5-labeled Oxi-αCD NPs and FA-Oxi-αCD NPs in a murine model of CBP. **(A)** *In vivo* fluorescence images of **(I)** saline, (II) free Cy5, (III) Cy5-labeled Oxi-αCD NPs and (IV) Cy5-labeled FA-Oxi-αCD in a murine model of CBP at different time points after intravenous injection (the region in the yellow circle indicates the lower urinary tract and part of the reproductive system). **(B–C)** *Ex vivo* fluorescence images of the excised prostates at 24 h **(B)** and 48 h **(C)** postinjection: (a) saline, (b) free Cy5, (c) Cy5-labeled Oxi-αCD NPs (150–160 nm), (d) Cy5-labeled FA-Oxi-αCD NPs (150–160 nm), (e) Cy5-labeled Oxi-αCD NPs (180–190 nm), (f) Cy5-labeled FA-Oxi-αCD NPs (180–190 nm), (g) Cy5-labeled Oxi-αCD NPs (>220 nm) and (h) Cy5-labeled FA-Oxi-αCD NPs (>220 nm). **(D–H)** ROI analysis of fluorescence intensity in the lower urinary tract and part of the reproductive system was performed at different time points after intravenous injection (n = 3 per group). **(I)** ROI analysis of the fluorescence intensity of the excised prostate at 24 h and 48 h *, significantly different at *p* < 0.05; **, significantly different at *p* < 0.01; ***, significantly different at *p* < 0.001; ****, significantly different at *p* < 0.0001.

The fluorescence intensity of CBP mice treated with free Cy5 decreased significantly with the extension of observation time, especially after 2 h of injection, indicating that Cy5 dyes were quickly cleared from the body. However, both Cy5-labeled Oxi-αCD NPs and FA-Oxi-αCD NPs showed higher fluorescence signals in the lower urinary tract and parts of the reproductive system than free Cy5 after 4, 8 and 24 h of administration, whereas the 180–190 nm group still maintained obvious fluorescence intensity at 48 h (Figure 3A). Semiquantitative analysis indicated that mice that received Cy5-labeled FA-Oxi-αCD NPs in the 180–190 nm group exhibited stronger fluorescence intensity in the lower urinary tract and parts of the reproductive system of CBP mice at all five observation time points compared to other NPs in the 150–160 nm group and >220 nm group (Figures 3D–H).

To further investigate the targeting efficiency of NPs to prostate tissues in CBP mice, the isolated prostate tissues were subjected to *ex vivo* imaging at 24 and 48 h of administration. Bright fluorescence intensity was observed in the prostate tissues of the NPs group, which indicated that their accumulation in prostate tissues was increased, and the fluorescence signals of CBP mice treated with Cy5-labeled FA-Oxi-αCD NPs remained stronger than that of CBP mice treated with Cy5-labeled Oxi-αCD NPs at 48 h postinjection (Figures 3B,C). Our previous work demonstrated that overexpression of FRs was detected in prostate tissues of CBP mice, which favors the specific binding of FA-modified NPs to FRs in prostate tissues. Therefore, this promoted the accumulation of FA-modified NPs in prostate tissues, which was higher than that of nontargeted NPs. In accordance with the *in vivo* imaging results, Cy5-labeled FA-Oxi-αCD NPs in the 180–190 nm group displayed the highest fluorescence signals in prostate tissues than the other NPs (Figure 3I), suggesting that FA-modified Oxi-αCD NPs with a size of 180–190 nm had longer blood circulation and a better targeting ability. These NPs are relatively suitable as a nanodrug platform for the targeted delivery of anti-inflammatory agents to prostate tissues. In addition, the basic cell experiments and biosafety of FA-modified Oxi-αCD NPs approximately 180–190 nm were verified in our previous study (Zheng et al., 2022).

**FIGURE 4:**
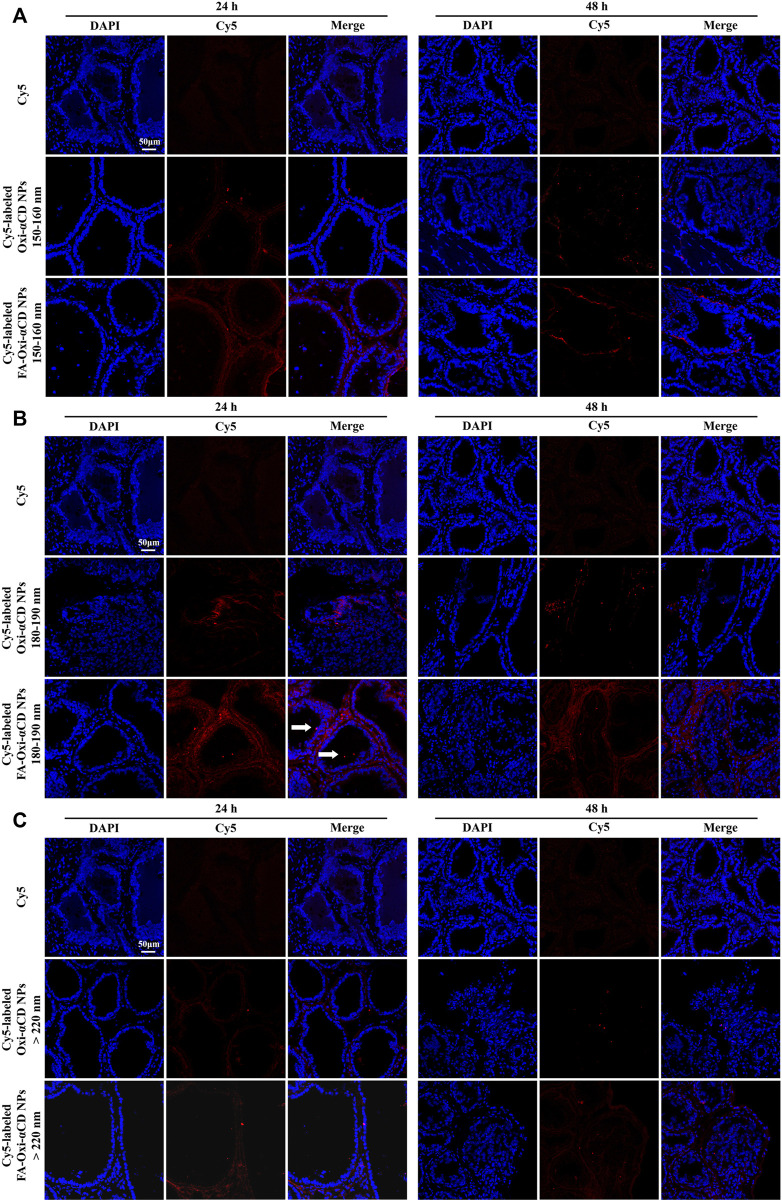
CLSM images of prostate tissues treated with Cy5 or Cy5-labeled NPs for 24 and 48 h. Red indicates NPs, and blue indicates DAPI. **(A)** 150–160 nm, **(B)** 180–190 nm and **(C)** > 220 nm. The scale bar represents 50 μm.

Furthermore, frozen sections of the excised prostate tissues were collected to observe and analyze which NPs can better penetrate prostate tissues. The frozen section results also demonstrated that Cy5-labeled FA-Oxi-αCD NPs could effectively accumulate in the prostate tissues (Figure 4). Consistent with the previous conclusion, Cy5-labeled FA-Oxi-αCD NPs in the 180–190 nm group demonstrated maximum accumulation in the prostate tissues, and some fluorescence signals were found in the prostate lumen (Figure 4B), indicating that they had the potential to deliver drugs to the interior of prostate tissues.

### 3.3 *In vivo* biodistribution of cRGD-modified NPs with different particle sizes

As mentioned above, NPs modified with cRGD peptide, a ligand of α_v_β_1_ integrin receptor, are expected to achieve efficient recognition of neutrophils, thereby improving the function of active targeting to prostate tissues. Therefore, we explored the targeting ability of cRGD-modified NPs to inflammatory prostate tissues and discussed the effect of particle size on the targeting efficiency.

**FIGURE 5:**
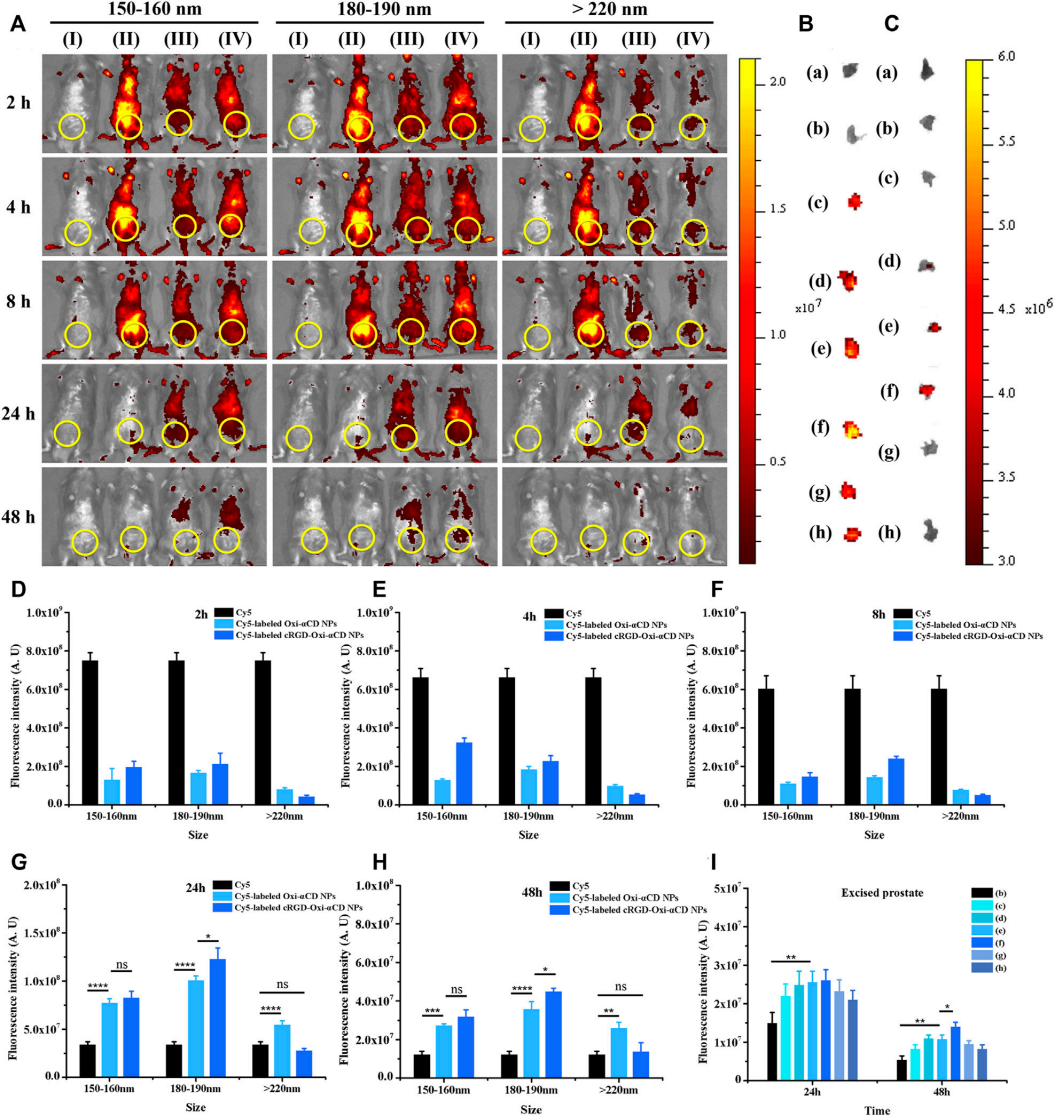
*In vivo* distribution of Cy5-labeled Oxi-αCD NPs and RGD-Oxi-αCD NPs in a murine model of CBP. **(A)** *In vivo* fluorescence images of **(I)** saline, (II) free Cy5, (III) Cy5-labeled Oxi-αCD NPs and (IV) Cy5-labeled RGD-Oxi-αCD in a murine model of CBP at different time points after intravenous injection (the region in the yellow circle indicates the lower urinary tract and part of the reproductive system). **(B–C)** *Ex vivo* fluorescence images of the excised prostates at 24 h **(B)** and 48 h **(C)** postinjection: (a) saline, (b) free Cy5, (c) Cy5-labeled Oxi-αCD NPs (150–160 nm), (d) Cy5‐labeled cRGD‐Oxi‐αCD NPs (150‐160 nm), (e) Cy5‐labeled Oxi‐αCD NPs (180‐190 nm), (f) Cy5‐labeled cRGD‐Oxi‐αCD NPs (180‐190 nm), (g) Cy5‐labeled Oxi‐αCD NPs (>220 nm) and (h) Cy5‐labeled cRGD‐Oxi‐αCD NPs (>220 nm). **(D–H)** ROI analysis of fluorescence intensity in the lower urinary tract and part of the reproductive system was performed at different time points after intravenous injection (*n* = 3 per group). **(I)** ROI analysis of the fluorescence intensity of the excised prostate at 24 h and 48 h *, significantly different at *p* < 0.05; **, significantly different at *p* < 0.01; ***, significantly different at *p* < 0.001; ****, significantly different at *p* < 0.0001.

Compared with the control group, only weak fluorescence intensity was observed in the lower urinary tract and parts of the reproductive system of CBP mice treated with free Cy5 at 24 and 48 h postinjection, indicating that few Cy5 dyes aggregated at the sites of prostatitis. In contrast, an obvious fluorescence signal was still detected in CBP mice treated with NPs in the 180–190 nm group, suggesting that the retention time of these NPs in the body was longer than that of free Cy5 (Figure 5A). Semiquantitative analysis also proved that mice that received Cy5-labeled cRGD-Oxi-αCD NPs in the 180–190 nm group exhibited higher fluorescence intensity in the lower urinary tract and parts of the reproductive system of CBP mice after 24 and 48 h of administration than other NPs in the 150–160 group and >220 nm group (Figures 5D–H).

In the same way, *ex vivo* prostate tissue imaging was performed at 24 and 48 h to further study the targeting ability of NPs on CBP mouse prostate tissues. After 24 h of injection, the prostate tissues of each group showed bright fluorescence signals compared with the free Cy5 group. However, only the fluorescence intensity of CBP mice treated with NPs in the 180–190 nm group retained a significant fluorescence signal at 48 h postinjection (Figures 5B,C). Moreover, Cy5-labeled cRGD-Oxi-αCD NPs in the 180–190 nm group displayed the highest fluorescence signals in prostate tissues (Figure 5I), which was in accordance with the *in vivo* imaging results, indicating that cRGD modification could enhance the accumulation of NPs in inflammatory prostate tissues.

**FIGURE 6:**
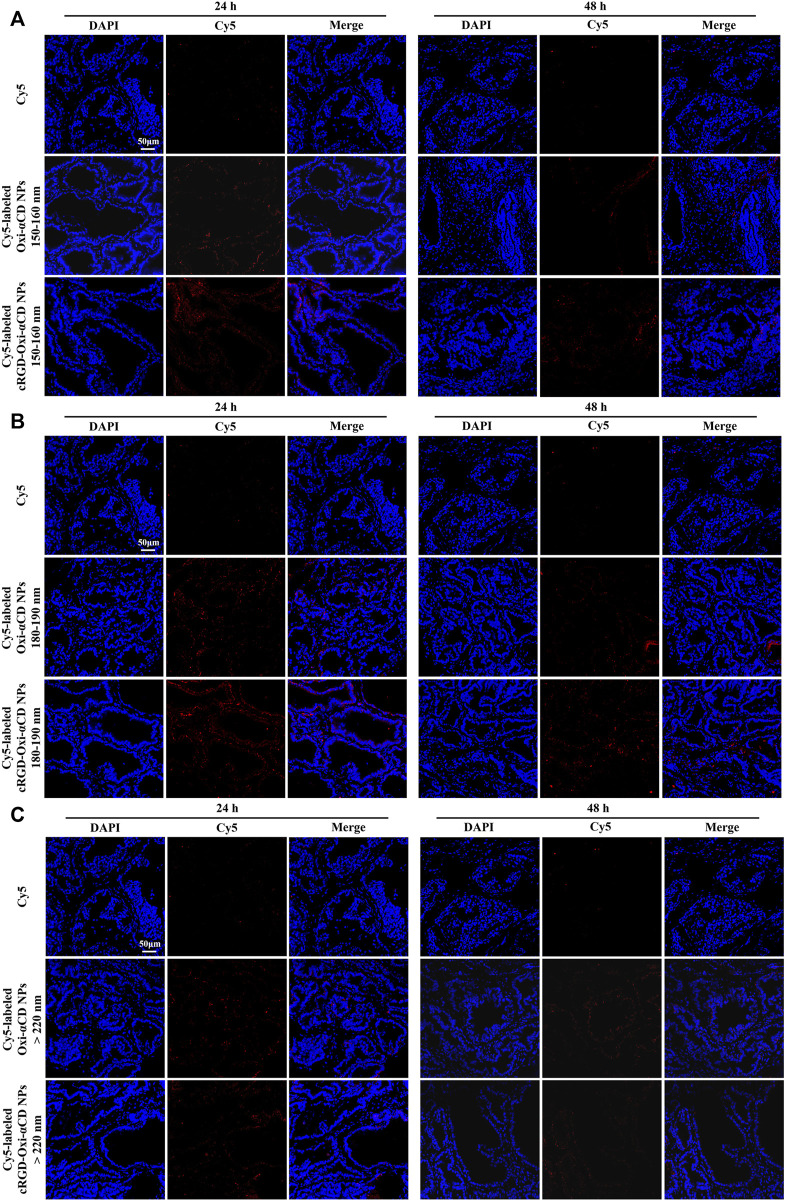
CLSM images of prostate tissues treated with Cy5 or Cy5-labeled NPs for 24 and 48 h. Red indicates NPs, and blue indicates DAPI. **(A)** 150–160 nm, **(B)** 180–190 nm and **(C)** > 220 nm. The scale bar represents 50 μm.

Frozen sections of the excised prostate tissues also demonstrated that Cy5-labeled NPs with particle sizes of 180–190 nm could effectively accumulate in the prostate tissues both after 24 and 48 h of injection, and the targeted NPs were superior to the nontargeted NPs (Figure 6B). However, the fluorescence of mice treated with Cy5-labeled NPs in the 150–160 nm group and >220 nm group almost disappeared at 48 h postinjection (Figures 6A,C). Based on the above results, similar to the conclusions discussed with FA-modified NPs, cRGD-modified NPs with particle sizes of 180–190 nm had a satisfactory targeting efficiency to prostate tissues.

### 3.4 Comparison of FA-modified and cRGD-modified NPs

The targeting ability of FA-modified and cRGD-modified NPs with different particle sizes was discussed above, and both showed the optimal targeting efficiency to inflammatory prostate tissues when the particle size was 180–190 nm. Their targeted penetration capabilities to prostate tissues at the same particle diameter were further explored.

**FIGURE 7:**
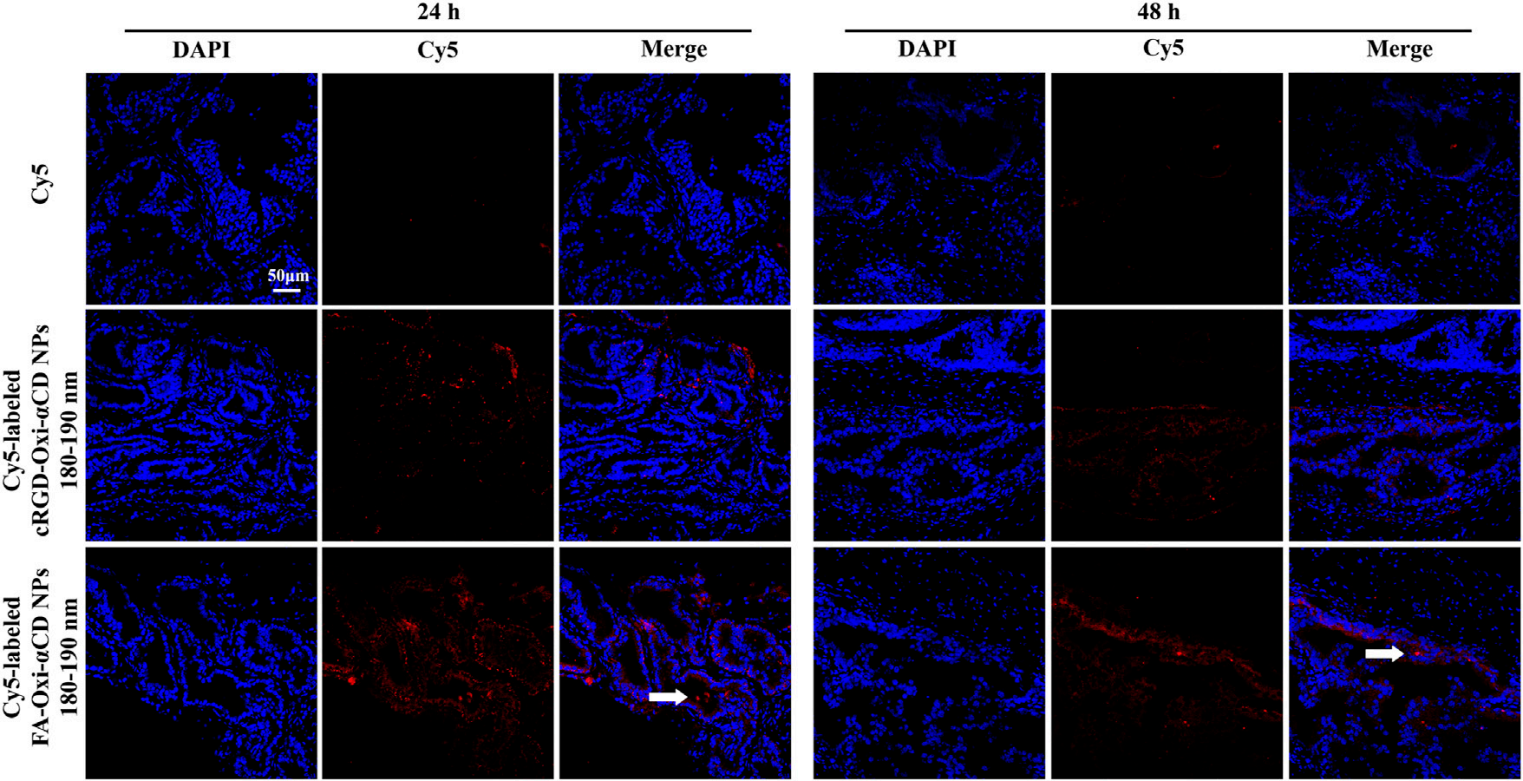
CLSM images of prostate tissues treated with Cy5, Cy5-labeled RGD-Oxi-αCD NPs (180–190 nm) and Cy5-labeled FA-Oxi-αCD NPs (180–190 nm) for 24 and 48 h. Red indicates NPs, and blue indicates DAPI. The scale bar represents 50 μm.

Through the frozen sections (Figure 7), it was observed that some fluorescence signals were found in the prostate lumen after the mice were treated with Cy5-labeled FA-Oxi-αCD NPs, suggesting that the FA-modified NPs could be effectively transferred to the prostate lumen and thus accumulated in the glandular lumen, which was superior to the mice treated with Cy5-labeled cRGD-Oxi-αCD NPs.

### 3.5 Comparison of different injection methods

Based on the discussion above, it was apparent to conclude that FA-modified NPs with a particle size of 180–190 nm were the optimal nanodrug delivery platform for targeting and penetrating prostate tissues. Since the mouse model of CBP was established by bacterial infection through the urethra, we also investigated the effects of two different injection modes, urethral injection and tail intravenous injection, on the targeting efficiency of the above optimal NPs to prostate tissues.

**FIGURE 8:**
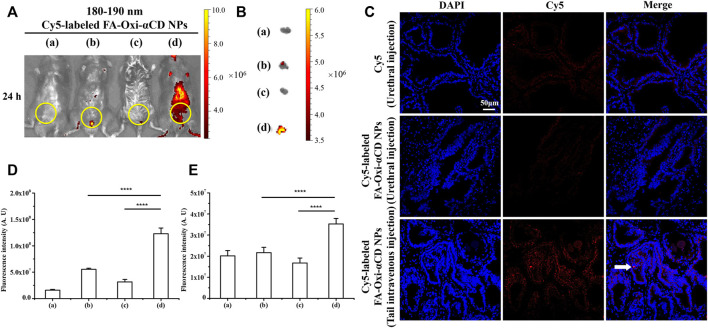
Effect of injection mode on NPs targeting inflammatory prostate tissue. **(A)** *In vivo* fluorescence images of (a) saline, (b) free Cy5 (Urethral injection), (c) Cy5-labeled FA-Oxi-αCD NPs (Urethral injection) and (d) Cy5-labeled FA-Oxi-αCD (Tail intravenous injection) in a murine model of CBP at 24 h after intravenous injection (the region in the yellow circle indicates the lower urinary tract and part of the reproductive system). **(B)** *Ex vivo* fluorescence images of the excised prostates at 24 h postinjection. **(C)** CLSM images of prostate tissues treated with Cy5 or Cy5-labeled NPs for 24 h. Red indicates NPs, and blue indicates DAPI. The scale bar represents 50 μm. **(D)** ROI analysis of fluorescence intensity in the lower urinary tract and part of the reproductive system was performed at 24 h after intravenous injection (n = 3 per group). **(E)** ROI analysis of the fluorescence intensity of the excised prostate at 24 h ****, significantly different at *p* < 0.0001.

CBP mice were treated with Cy5-labeled FA-Oxi-αCD NPs, 180–190 nm, by tail intravenous injection, and considerable fluorescence signals were observed at 24 h postinjection by living imaging assay, while the fluorescence signal was extremely weak by urethral injection (Figure 8A). The intensity of isolated prostate fluorescence in each group was similar to the *in vivo* imaging results (Figure 8B). According to semiquantitative results, the fluorescence intensity of mice injected with NPs through the tail vein was the strongest, and there was a statistically significant difference from other groups (Figures 8D,E). Frozen sections of prostate tissue were detected using CLSM, and it demonstrated that NPs via tail intravenous injection accumulated the most in prostate tissues (Figure 8C), revealing that intravenous injection expressed a preferable targeting efficiency compared with urethral injection.

## 4 Discussion

The therapeutic efficacy of CBP caused by bacterial infection is limited by the difficulty of free agents (e.g., antibiotics) penetrating the prostate epithelium and targeting inflammatory tissues. To address this question, we further investigated the performance of NPs targeting inflammatory prostate tissues on the basis of previous nanotechnology treatment of CBP (Zheng et al., 2022). Spherical NPs with different physicochemical properties have been successfully prepared to verify their targeting capabilities to inflammatory prostate tissues of CBP, including comparison of three particle sizes (150–160, 180–190 and >220 nm) and two different surface modification ligands (FA and cRGD peptide), and the differences between the two different injection methods (tail intravenous and urethral injection) were discussed.

*In vivo* and *ex vivo* imaging results confirmed that both FA-modified NPs and cRGD-modified NPs could enhance their accumulation in prostate tissues of CBP mice due to the high expression of FRs and increase in neutrophils in inflammatory prostate tissues, respectively. We found that 180–190 nm was the most suitable particle size range, while the other two groups remained in the body for less time, which was not conducive to long circulation and passive targeting. For example, in the >220 nm group, NPs modified with FA and cRGD peptide both showed almost the weakest fluorescence signals, which may be because NPs larger than 200 nm were easily cleared by the liver and spleen (Sunoqrot et al., 2014; Blanco et al., 2015; Fan et al., 2020). In addition, immune cells such as macrophages phagocytose foreign particles in a size-dependent manner, with larger particles being more likely to be phagocytosed by macrophages than smaller particles (Fang et al., 2006; Pacheco et al., 2013; Miyamoto et al., 2021). The active targeting and penetrating capabilities of FA-modified NPs to prostate tissues were superior to those of cRGD-modified NPs, and tail intravenous injection was beneficial for NPs to enter the blood circulation and achieve the purpose of targeting prostate tissues.

In summary, we studied and discussed the influencing factors of NPs targeting inflammatory prostate tissues, focusing on the effects of size and different modification ligands on the targeting performance, and proved that FA-modified NPs with particle sizes of 180–190 nm can perform preferable targeting efficiency via tail intravenous injection. The results provide a new experimental basis and theoretical support for the targeted treatment of prostate-related diseases with nanotechnology in the future.

## Data Availability

The original contributions presented in the study are included in the article/Supplementary Material, further inquiries can be directed to the corresponding authors.

## References

[B1] Ahn H. K. Koo K. C. Chung B. H. Lee K. S. (2018). Comparison of the delta neutrophil index with procalcitonin, erythrocyte sedimentation rate, and C-reactive protein as predictors of sepsis in patients with acute prostatitis. Prostate Int. 6, 157–161. 10.1016/j.prnil.2018.05.001 30505819 PMC6251943

[B2] Attia M. F. Anton N. Wallyn J. Omran Z. Vandamme T. F. (2019). An overview of active and passive targeting strategies to improve the nanocarriers efficiency to tumour sites. J. Pharm. Pharmacol. 71, 1185–1198. 10.1111/jphp.13098 31049986

[B3] Blanco E. Shen H. Ferrari M. (2015). Principles of nanoparticle design for overcoming biological barriers to drug delivery. Nat. Biotechnol. 33, 941–951. 10.1038/nbt.3330 26348965 PMC4978509

[B4] Cao F. Gui S. Y. Gao X. Zhang W. Fu Z. Y. Tao L. M. (2022). Research progress of natural product-based nanomaterials for the treatment of inflammation-related diseases. Mat. Des. 218, 110686. 10.1016/j.matdes.2022.110686

[B5] Cao J. Huang D. Peppas N. A. (2020). Advanced engineered nanoparticulate platforms to address key biological barriers for delivering chemotherapeutic agents to target sites. Adv. Drug Deliv. Rev. 167, 170–188. 10.1016/j.addr.2020.06.030 32622022

[B6] Charalabopoulos K. Karachalios G. Baltogiannis D. Charalabopoulos A. Giannakopoulos X. Sofikitis N. (2003). Penetration of antimicrobial agents into the prostate. Chemotherapy 49, 269–279. 10.1159/000074526 14671426

[B7] Dorschner R. A. Lee J. Cohen O. Costantini T. Baird A. Eliceiri B. P. (2020). ECRG4 regulates neutrophil recruitment and CD44 expression during the inflammatory response to injury. Sci. Adv. 6, eaay0518. 10.1126/sciadv.aay0518 32195341 PMC7065879

[B8] Duan X. Li Y. (2013). Physicochemical characteristics of nanoparticles affect circulation, biodistribution, cellular internalization, and trafficking. SMALL 9, 1521–1532. 10.1002/smll.201201390 23019091

[B9] Fan W. Yu Z. Peng H. He H. Lu Y. Qi J. (2020). Effect of particle size on the pharmacokinetics and biodistribution of parenteral nanoemulsions. Int. J. Pharm. X. 586, 119551. 10.1016/j.ijpharm.2020.119551 32565287

[B10] Fang C. Shi B. Pei Y. Y. Hong M. H. Wu J. Chen H. Z. (2006). *In vivo* tumor targeting of tumor necrosis factor-alpha-loaded stealth nanoparticles: Effect of MePEG molecular weight and particle size. Eur. J. Pharm. Sci. 27, 27–36. 10.1016/j.ejps.2005.08.002 16150582

[B11] Golombek S. K. May J.-N. Theek B. Appold L. Drude N. Kiessling F. (2018). Tumor targeting via EPR: Strategies to enhance patient responses. Adv. Drug Deliv. Rev. 130, 17–38. 10.1016/j.addr.2018.07.007 30009886 PMC6130746

[B12] Halabi J. Jagger B. W. Salazar V. Winkler E. S. White J. P. Humphrey P. A. (2020). Zika virus causes acute and chronic prostatitis in mice and macaques. J. Infect. Dis. 221, 1506–1517. 10.1093/infdis/jiz533 31616920 PMC7137895

[B13] Hou J. Yang X. Li S. Cheng Z. Wang Y. Zhao J. (2019). Accessing neuroinflammation sites: Monocyte/neutrophil-mediated drug delivery for cerebral ischemia. Sci. Adv. 5, eaau8301. 10.1126/sciadv.aau8301 31531392 PMC6737273

[B14] Hou M. Wu X. Zhao Z. Deng Q. Chen Y. Yin L. (2022). Endothelial cell-targeting, ROS-ultrasensitive drug/siRNA co-delivery nanocomplexes mitigate early-stage neutrophil recruitment for the anti-inflammatory treatment of myocardial ischemia reperfusion injury. Acta Biomater. 143, 344–355. 10.1016/j.actbio.2022.02.018 35189380

[B15] Izci M. Maksoudian C. Manshian B. B. Soenen S. J. (2021). The use of alternative strategies for enhanced nanoparticle delivery to solid tumors. Chem. Rev. 121, 1746–1803. 10.1021/acs.chemrev.0c00779 33445874 PMC7883342

[B16] Ma Y. Jiang K. Chen H. Shi Q. Liu H. Zhong X. (2022). Liquid exfoliation of V8C7 nanodots as peroxidase-like nanozymes for photothermal-catalytic synergistic antibacterial treatment. Acta Biomater. 149, 359–372. 10.1016/j.actbio.2022.06.031 35779771

[B17] Miyamoto H. Higuchi K. Nakashima Y. Fujiwara Y. Nakanishi Y. (2021). Culture system for a closer biological contact between macrophages and microparticles. Front. Mech. Eng. 7, 631128. 10.3389/fmech.2021.631128

[B18] Muhamad N. Plengsuriyakarn T. Na-Bangchang K. (2018). Application of active targeting nanoparticle delivery system for chemotherapeutic drugs and traditional/herbal medicines in cancer therapy: A systematic review. Int. J. Nanomedicine 13, 3921–3935. 10.2147/IJN.S165210 30013345 PMC6038858

[B19] Ni R. Song G. Fu X. Song R. Li L. Pu W. (2020). Reactive oxygen species-responsive dexamethasone-loaded nanoparticles for targeted treatment of rheumatoid arthritis via suppressing the iRhom2/TNF-α/BAFF signaling pathway. Biomaterials 232, 119730. 10.1016/j.biomaterials.2019.119730 31918224

[B20] Pacheco P. White D. Sulchek T. (2013). Effects of microparticle size and fc density on macrophage phagocytosis. PLoS One 8, e60989. 10.1371/journal.pone.0060989 23630577 PMC3632606

[B21] Pearce A. K. O’Reilly R. K. (2019). Insights into active targeting of nanoparticles in drug delivery: Advances in clinical studies and design considerations for cancer nanomedicine. Bioconjug. Chem. 30, 2300–2311. 10.1021/acs.bioconjchem.9b00456 31441642

[B22] Poh S. Chelvam V. Ayala-Lopez W. Putt K. S. Low P. S. (2018). Selective liposome targeting of folate receptor positive immune cells in inflammatory diseases. Nanomedicine Nanotechnol. Biol. Med. 14, 1033–1043. 10.1016/j.nano.2018.01.009 29410110

[B23] Safari H. Kelley W. J. Saito E. Kaczorowski N. Carethers L. Shea L. D. (2020). Neutrophils preferentially phagocytose elongated particles-An opportunity for selective targeting in acute inflammatory diseases. Sci. Adv. 6, eaba1474. 10.1126/sciadv.aba1474 32577517 PMC7286665

[B24] Santharam M. A. Khan F. U. Naveed M. Ali U. Ahsan M. Z. Khongorzul P. (2019). Interventions to chronic prostatitis/Chronic pelvic pain syndrome treatment. Where are we standing and what’s next? Eur. J. Pharmacol. 857, 172429. 10.1016/j.ejphar.2019.172429 31170381

[B25] Seo M.-Y. Im S.-J. Gu N.-Y. Kim J.-H. Chung Y.-H. Ahn M.-H. (2014). Inflammatory response of prostate epithelial cells to stimulation byTrichomonas vaginalis. Prostate 74, 441–449. 10.1002/pros.22766 24339030

[B26] Sfanos K. S. Yegnasubramanian S. Nelson W. G. De Marzo A. M. (2018). The inflammatory microenvironment and microbiome in prostate cancer development. Nat. Rev. Urol. 15, 11–24. 10.1038/nrurol.2017.167 29089606

[B27] Shukla S. K. Sarode A. Kanabar D. D. Muth A. Kunda N. K. Mitragotri S. (2021). Bioinspired particle engineering for non-invasive inhaled drug delivery to the lungs. Mater. Sci. Eng. C 128, 112324. 10.1016/j.msec.2021.112324 PMC8417427 34474875

[B28] Su Z. T. Zenilman J. M. Sfanos K. S. Herati A. S. (2020). Management of chronic bacterial prostatitis. Curr. Urol. Rep. 21, 29. 10.1007/s11934-020-00978-z 32488742

[B29] Sunoqrot S. Bugno J. Lantvit D. Burdette J. E. Hong S. (2014). Prolonged blood circulation and enhanced tumor accumulation of folate-targeted dendrimer-polymer hybrid nanoparticles. J. Control. RELEASE 191, 115–122. 10.1016/j.jconrel.2014.05.006 24837188 PMC4156894

[B30] Wang X. Fan L. Cheng L. Sun Y. Wang X. Zhong X. (2020). Biodegradable nickel disulfide nanozymes with GSH-depleting function for high-efficiency photothermal-catalytic antibacterial therapy. iScience 23, 101281. 10.1016/j.isci.2020.101281 32622263 PMC7334425

[B31] Wang X. Shi Q. Zha Z. Zhu D. Zheng L. Shi L. (2021a). Copper single-atom catalysts with photothermal performance and enhanced nanozyme activity for bacteria-infected wound therapy. Bioact. Mat. 6, 4389–4401. 10.1016/j.bioactmat.2021.04.024 PMC8111038 33997515

[B32] Wang X. Zhong X. Li J. Liu Z. Cheng L. (2021b). Inorganic nanomaterials with rapid clearance for biomedical applications. Chem. Soc. Rev. 50, 8669–8742. 10.1039/d0cs00461h 34156040

[B33] Wang Y. Wang Q. Feng W. Yuan Q. Qi X. Chen S. (2021c). Folic acid-modified ROS-responsive nanoparticles encapsulating luteolin for targeted breast cancer treatment. Drug Deliv. (Lond). 28, 1695–1708. 10.1080/10717544.2021.1963351 PMC8428179 34402706

[B34] Wang Y. Yuan Q. Feng W. Pu W. Ding J. Zhang H. (2019). Targeted delivery of antibiotics to the infected pulmonary tissues using ROS-responsive nanoparticles. J. Nanobiotechnology 17, 103. 10.1186/s12951-019-0537-4 31581948 PMC6777033

[B35] Wu K. Zhu D. Dai X. Wang W. Zhong X. Fang Z. (2022). Bimetallic oxide Cu1.5Mn1.5O4 cage-like frame nanospheres with triple enzyme-like activities for bacterial-infected wound therapy. Nano Today 43, 101380. 10.1016/j.nantod.2022.101380

[B36] Xiong S. Liu X. Deng W. Zhou Z. Li Y. Tu Y. (2020). Pharmacological interventions for bacterial prostatitis. Front. Pharmacol. 11, 504. 10.3389/fphar.2020.00504 32425775 PMC7203426

[B37] Yang Y. Guo L. Wang Z. Liu P. Liu X. Ding J. (2021). Targeted silver nanoparticles for rheumatoid arthritis therapy via macrophage apoptosis and Re-polarization. Biomaterials 264, 120390. 10.1016/j.biomaterials.2020.120390 32980634

[B38] Yoo J. Park C. Yi G. Lee D. Koo H. (2019). Active targeting strategies using biological ligands for nanoparticle drug delivery systems. Cancers (Basel). 11, 640. 10.3390/cancers11050640 PMC6562917 31072061

[B39] Zhang D. Wei Y. Chen K. Zhang X. Xu X. Shi Q. (2015). Biocompatible reactive oxygen species (ROS)-Responsive nanoparticles as superior drug delivery vehicles. Adv. Healthc. Mat. 4, 69–76. 10.1002/adhm.201400299 25147049

[B40] Zhao Z. Ukidve A. Krishnan V. Mitragotri S. (2019). Effect of physicochemical and surface properties on *in vivo* fate of drug nanocarriers. Adv. Drug Deliv. Rev. 143, 3–21. 10.1016/j.addr.2019.01.002 30639257

[B41] Zheng J. Hu R. Yang Y. Wang Y. Wang Q. Xu S. (2022). Antibiotic-loaded reactive oxygen species-responsive nanomedicine for effective management of chronic bacterial prostatitis. Acta Biomater. 143, 471–486. 10.1016/j.actbio.2022.02.044 35259516

