# Supplementary figures and images for: In vivo targeting capacities of different nanoparticles to prostate tissues based on a mouse model of chronic bacterial prostatitis
Source: Front Bioeng Biotechnol. 2022 Oct 6;10:1021385. doi: 10.3389/fbioe.2022.1021385 (PMC9582453; doi:10.3389/fbioe.2022.1021385)

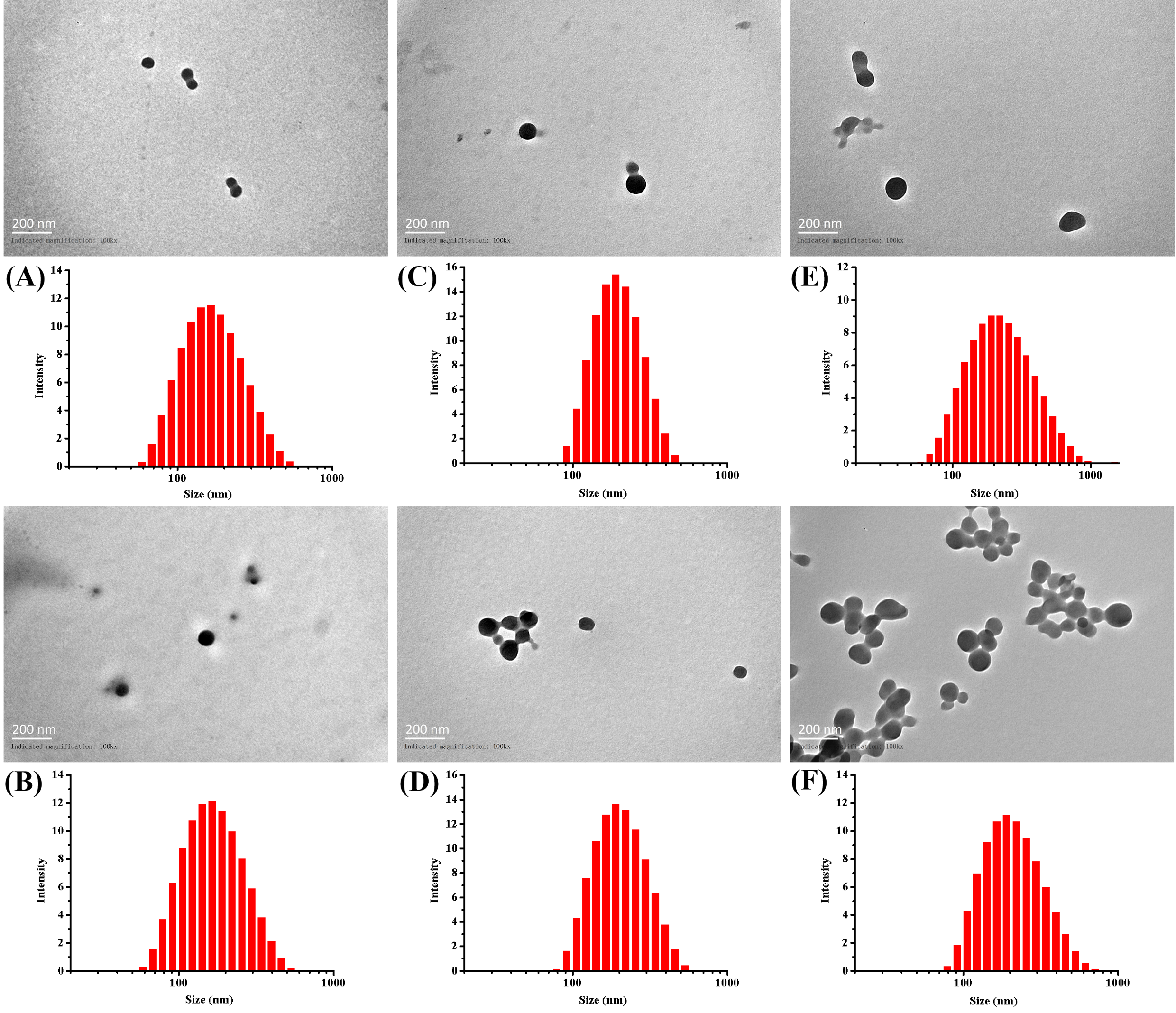

Supplement: Supplementary file 1 [file DataSheet1.ZIP › Figures/Fig1.tif]

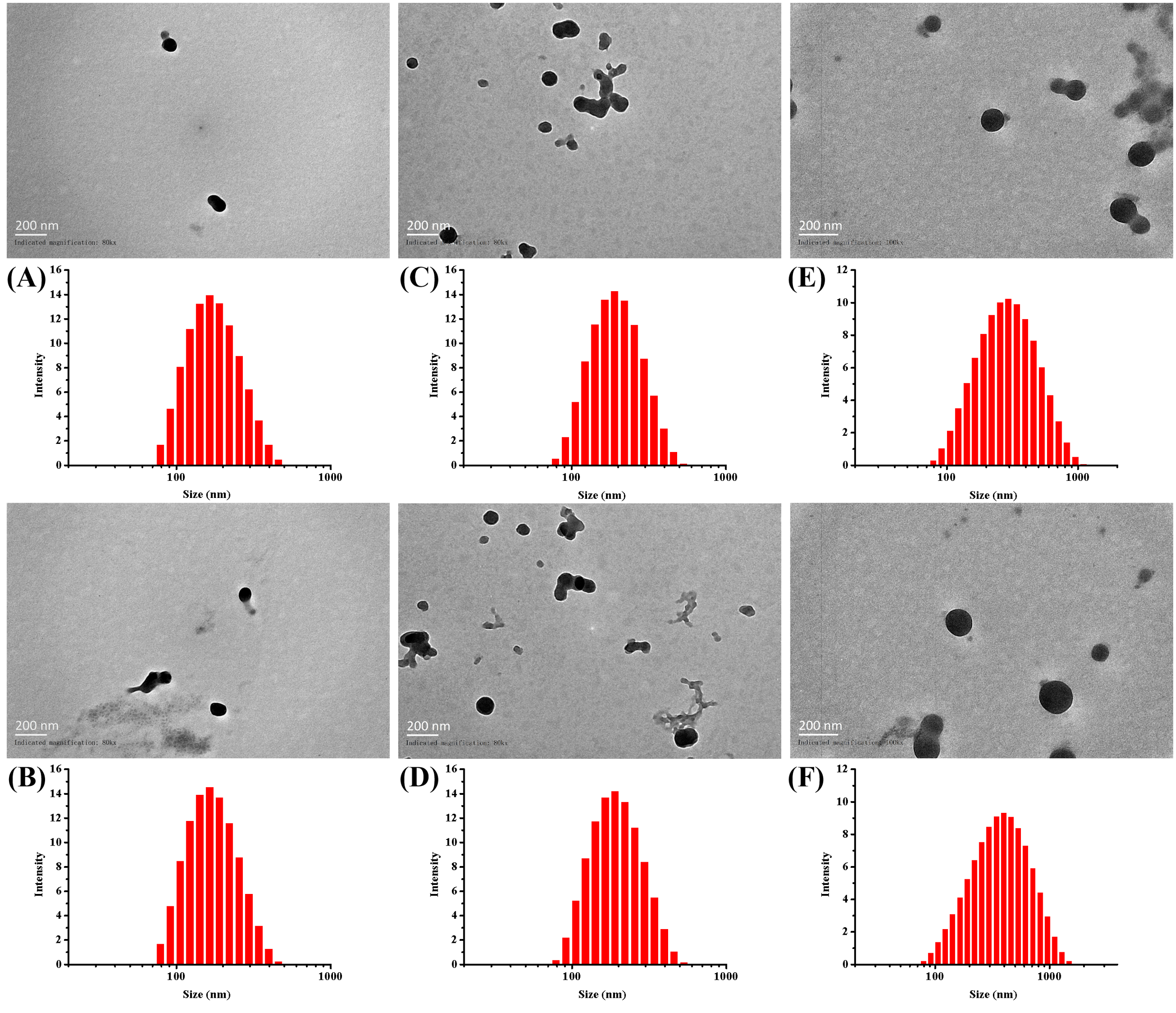

Supplement: Supplementary file 1 [file DataSheet1.ZIP › Figures/Fig2.tif]

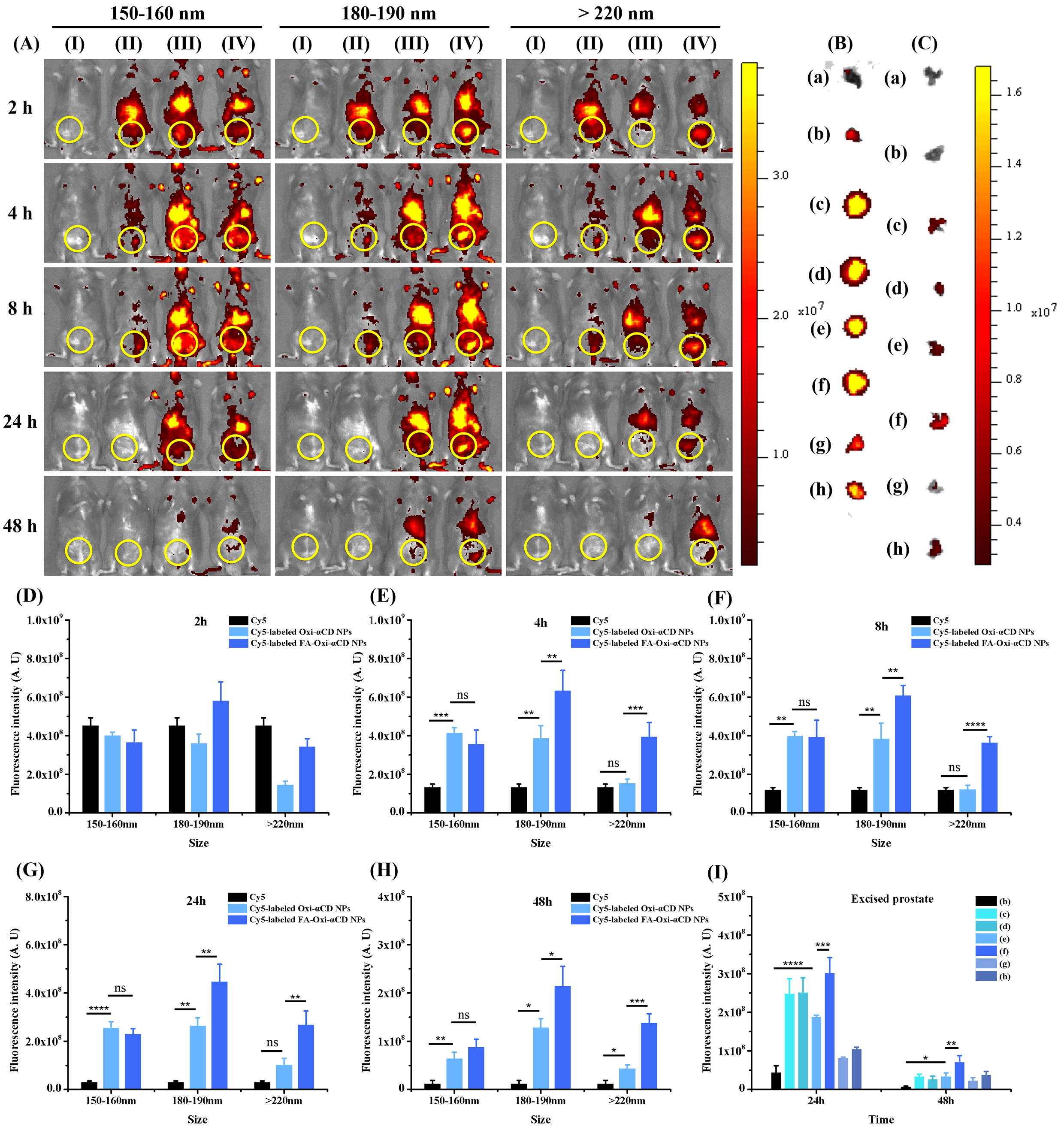

Supplement: Supplementary file 1 [file DataSheet1.ZIP › Figures/Fig3.tif]

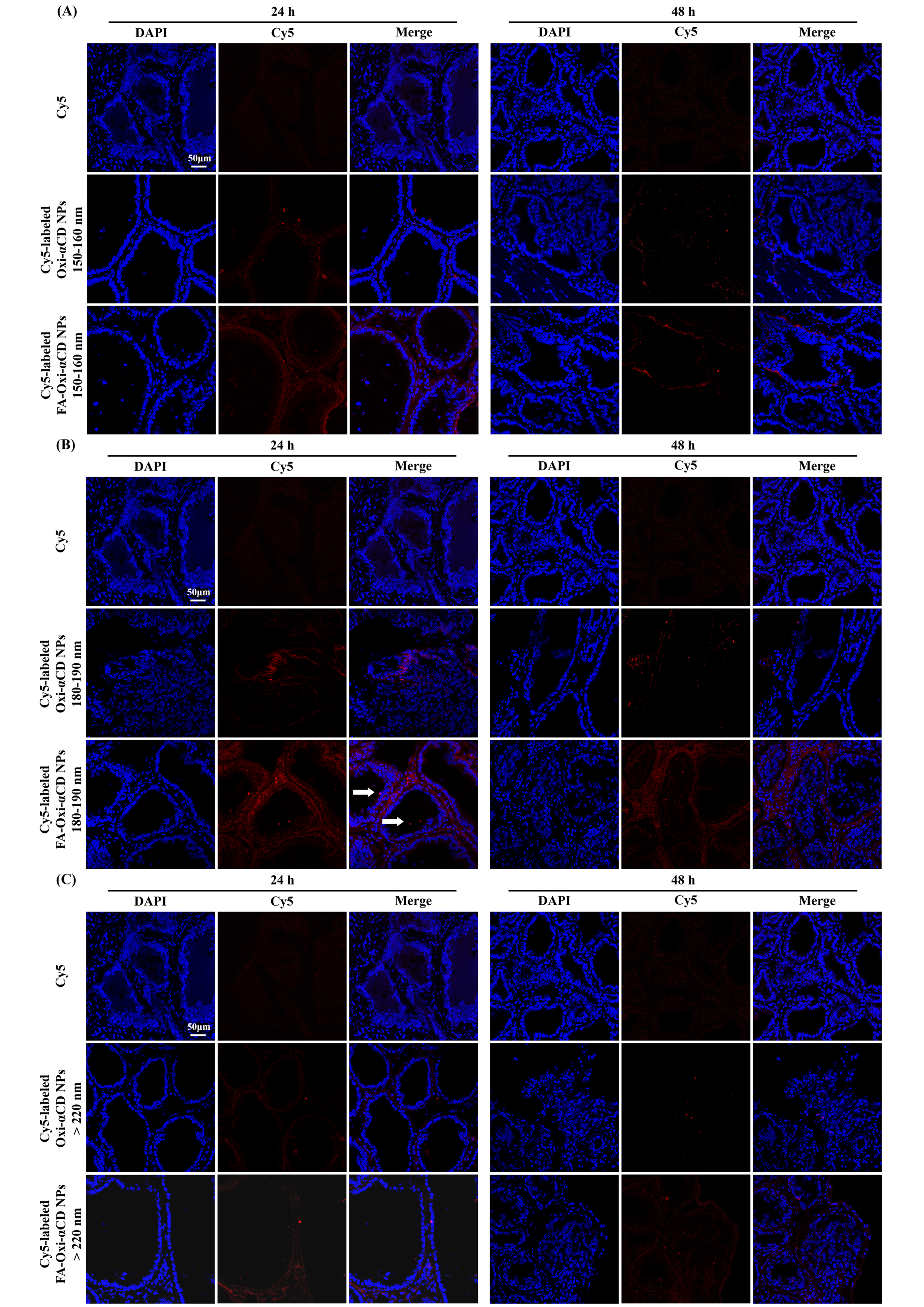

Supplement: Supplementary file 1 [file DataSheet1.ZIP › Figures/Fig4.tif]

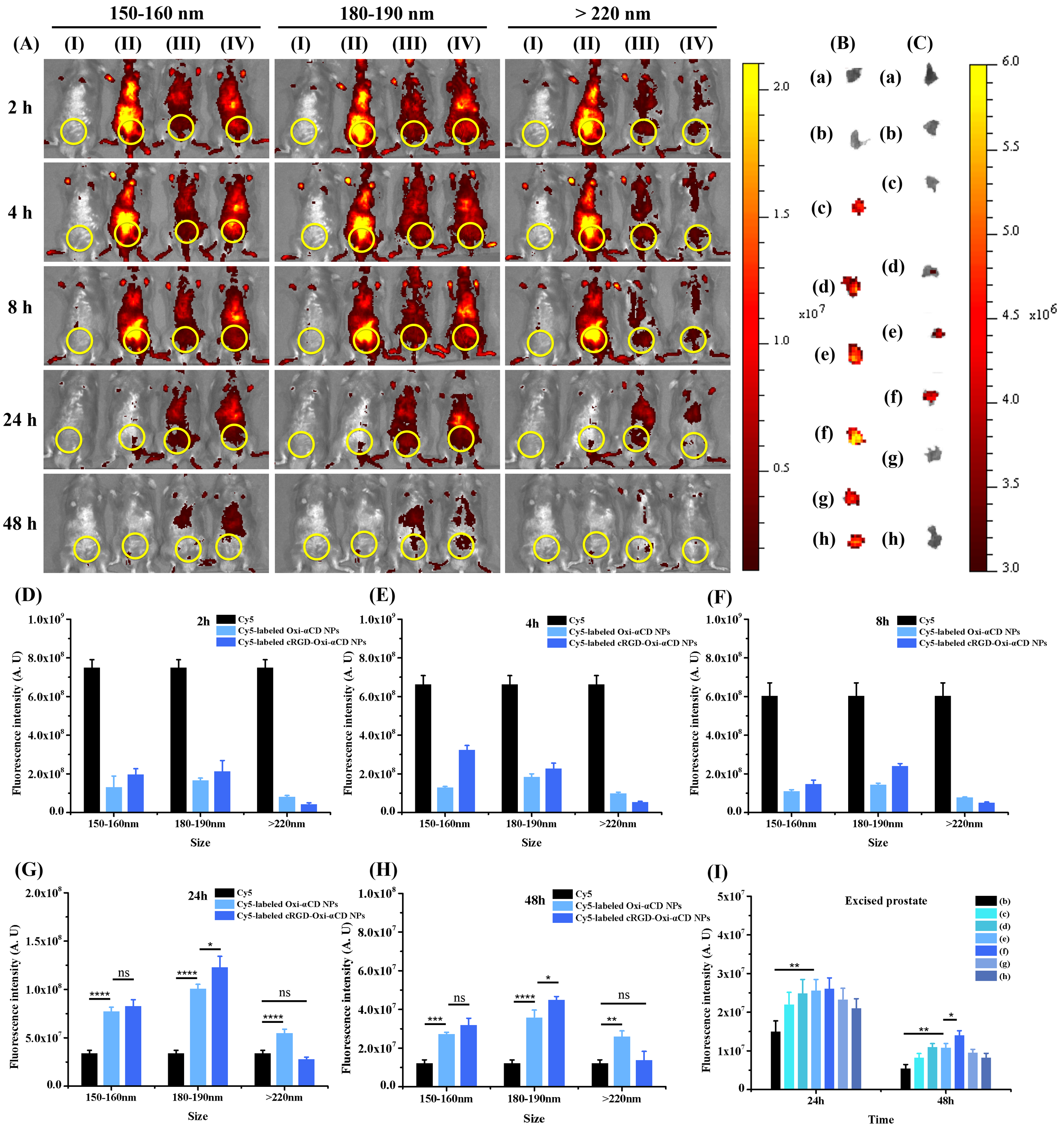

Supplement: Supplementary file 1 [file DataSheet1.ZIP › Figures/Fig5.tif]

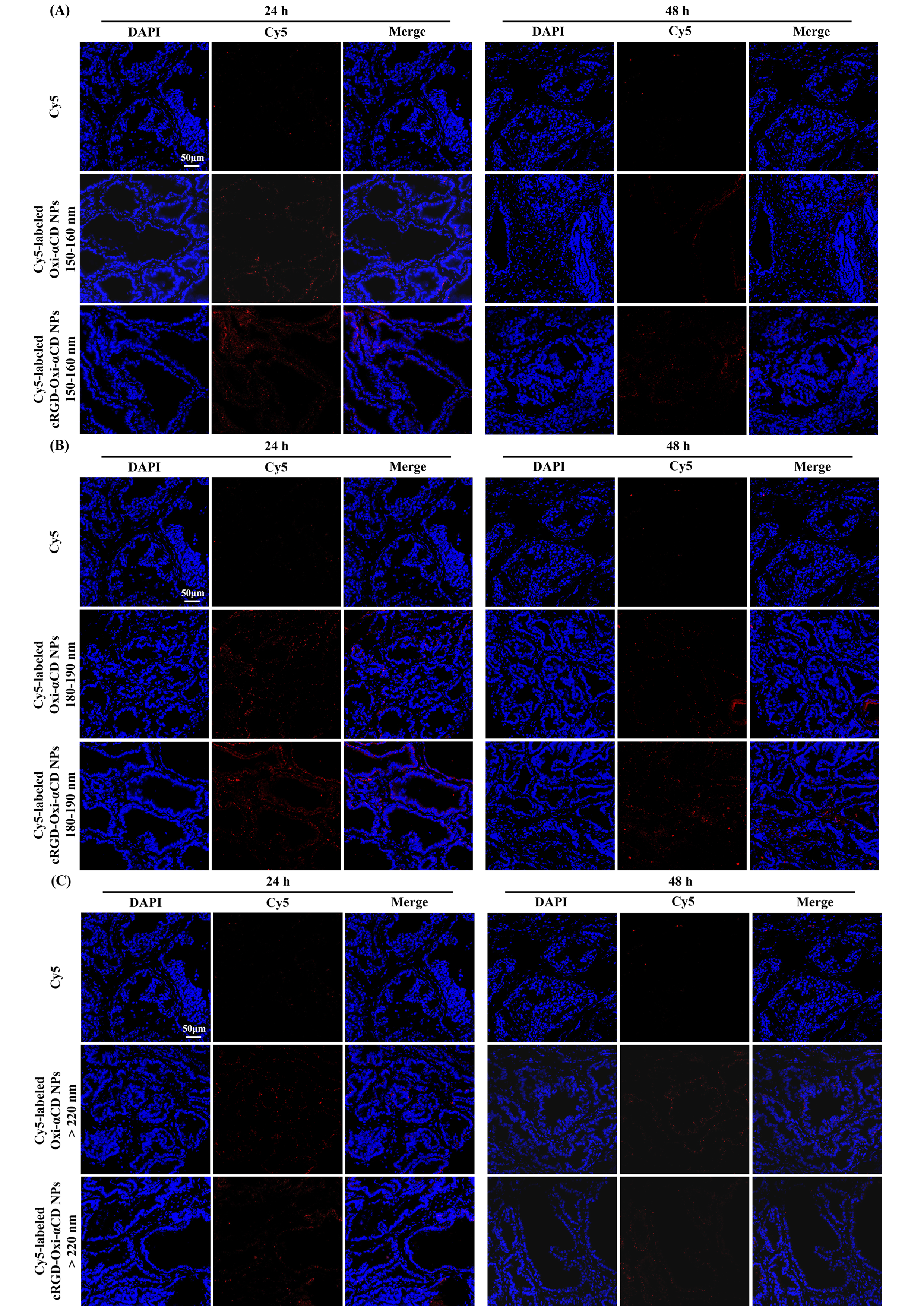

Supplement: Supplementary file 1 [file DataSheet1.ZIP › Figures/Fig6.tif]

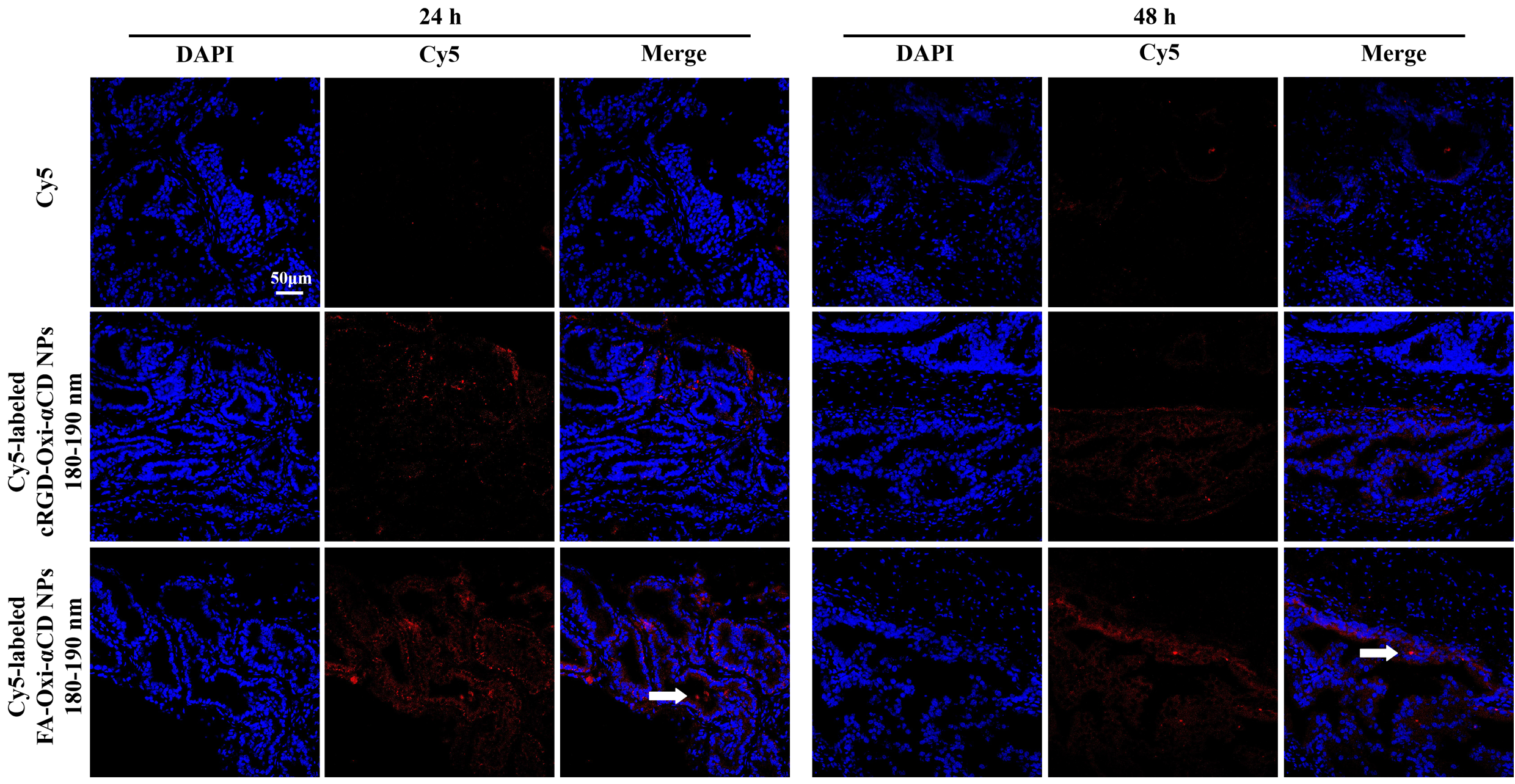

Supplement: Supplementary file 1 [file DataSheet1.ZIP › Figures/Fig7.tif]

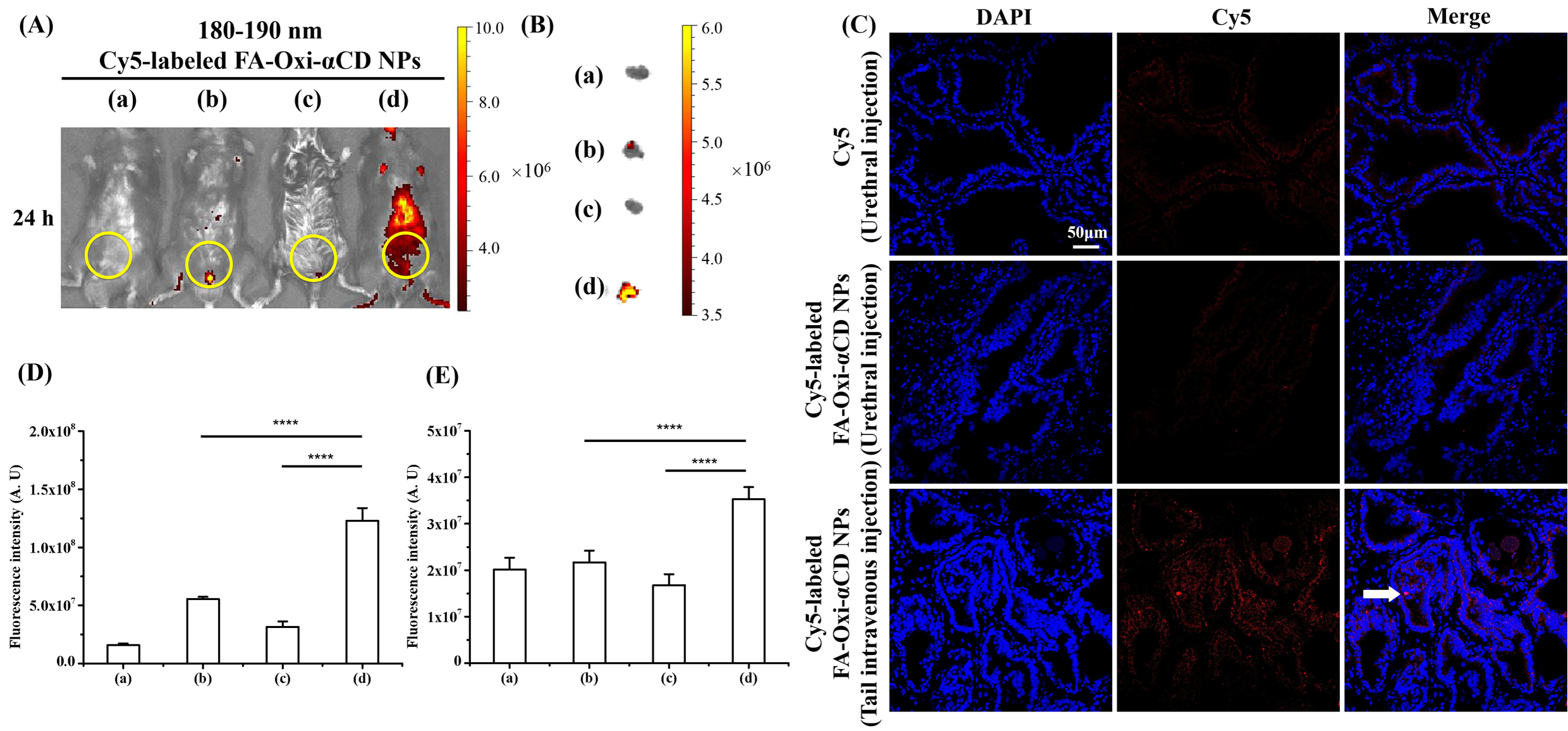

Supplement: Supplementary file 1 [file DataSheet1.ZIP › Figures/Fig8.tif]

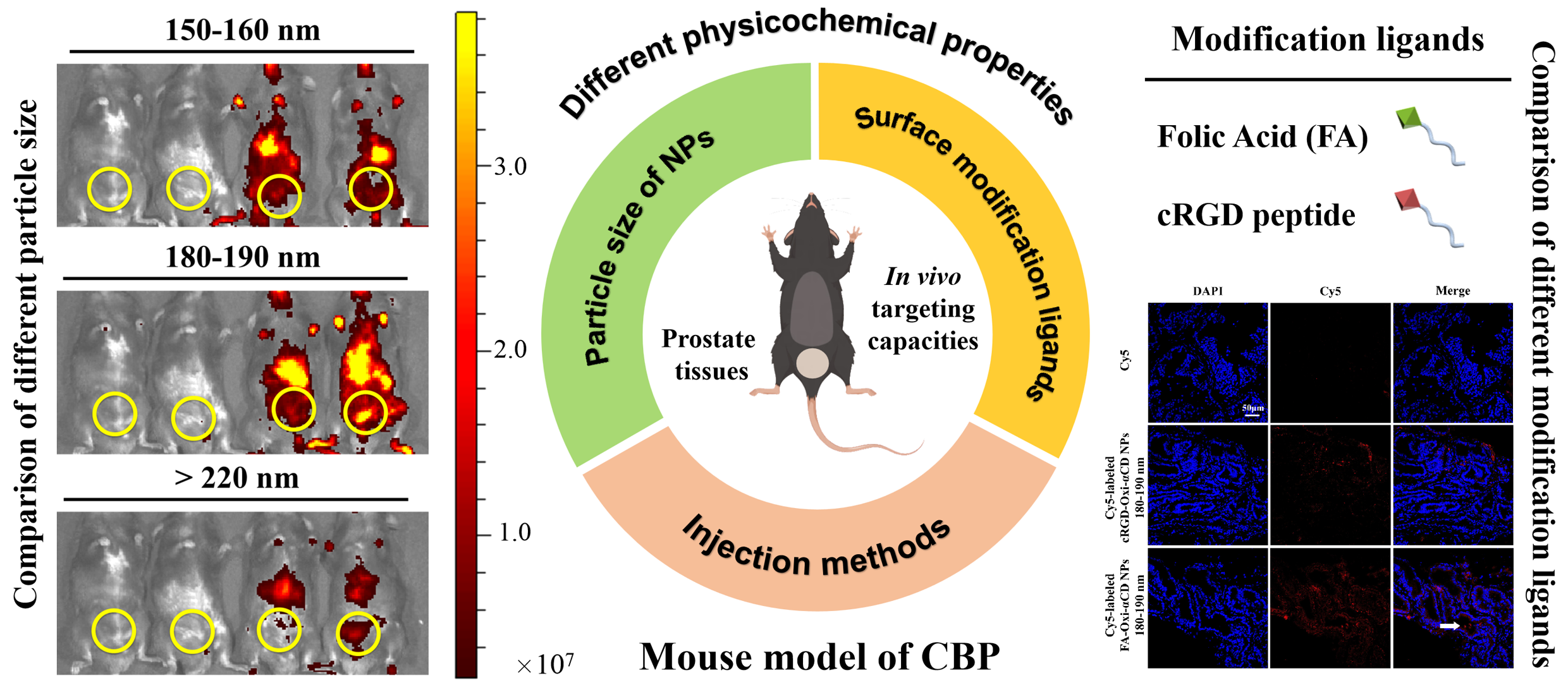

Supplement: Supplementary file 1 [file DataSheet1.ZIP › Figures/Graphic abstract.tif]
